# Supplementary material for: Rats adopt the optimal timescale for evidence integration in a dynamic environment
Source: Nat Commun. 2018 Oct 15;9:4265. doi: 10.1038/s41467-018-06561-y (PMC6189050; doi:10.1038/s41467-018-06561-y)
Supplement: Supplementary file 1 — Supplementary Information [file 41467_2018_6561_MOESM1_ESM.pdf]

Supplementary Materials - Rats adopt the optimal timescale for evidence integration in a dynamic environment

Piet et al.

Supplementary materials

The supplementary materials contains extended figures and control analyses for several aspects of the study:

1. Supplementary Note 1 - Training procedure
2. Supplementary Note 2 - Individual rat behavior
3. Supplementary Note 3 - Optimal inference derivation
4. Supplementary Note 4 - Decreasing click reliability lengthens integration timescales
5. Supplementary Note 5 - Click mislocalization from model parameters
6. Supplementary Note 6 - Click mislocalization is reliable across a wide range of click rates
7. Supplementary Note 7 - Alternative sensory noise models
8. Supplementary Note 8 - Linear approximation details
9. Supplementary Note 9 - Psychophysical reverse correlation details
10. Supplementary Note 10 - Ruling out last click strategies
11. Supplementary Note 11 - Ruling out poor estimates of environmental volatility
12. Supplementary Note 12 - Trial-by-trial model details
13. Supplementary Note 13 - Individual Rats in different environments

Supplementary Note 1 - Training procedure

The training process for the Dynamic Clicks task involves "classical" training during which the rats learn to associate ports with rewards. The "classical" pipeline is the same as Ref. [1]. After completing classical training, then rats then learn the standard Poisson clicks task as described in Ref. [2]. Finally, environmental state switching is introduced. The following tables outline keep changes at each stage in the procedure. Rats typically spend a few days on classical, and about 6 weeks moving through the clicks training.

Classical Training:

	Stage Name
1	learn left poking
2	learn right poking
3	learn center poke switching blocks

Dynamic Clicks Training:

	Stage Name	h	γ	T	ΔT
1	grow nose in center 1	0	5	1	0
2	wait for good endpoints	0	5	1	0
3	grow nose in center 2	0	5	2	0
4	wait for good endpoints	0	5	2	0
5	add variable trial length	0	5	2	0.5
6	wait for good endpoints	0	5	2	0.5
7	step hazard 1	1 Hz	5	2	0.5
9	increase variable trial length	1	5	2	1.5
10	add psychometrics 1	1	4	2	1.5
11	add psychometrics 2	1	3.5	2	1.5
12	add psychometrics 3	1	3.2	2	1.5
13	add psychometrics 4	1	3	2	1.5
14	final	1	3	2	1.5

Notes: **h** is the hazard rate between states. H is introduced in stage 9, increased in stage 10, and is constant afterwards. **Gamma** (γ) is the log of the click rates. $\gamma = 5$ is the setting for endpoints, which is extremely easy. **T** is the maximum duration of the stimulus period. ΔT is the variability in stimulus period duration. If the trial length (T) is 2 seconds, and ΔT is 0.5, that means trials are 1.5 - 2 seconds in length.

Supplementary Note 2 - Individual rat behavior

In this section we outline our rat training process, and then provide several model free analyses of behavior for each rat individually. First, we include the psychometric curve with respect to the total click difference on each trial. Second, we include the psychometric curve with respect to the optimal inference process (assuming no sensory noise). Third, the chronometric plot shows rat accuracy with respect to time since the last hidden state change. Fourth, the chronometric plot with respect to total trial duration. Fifth, the reverse correlation curves with best fit exponential for each rat.

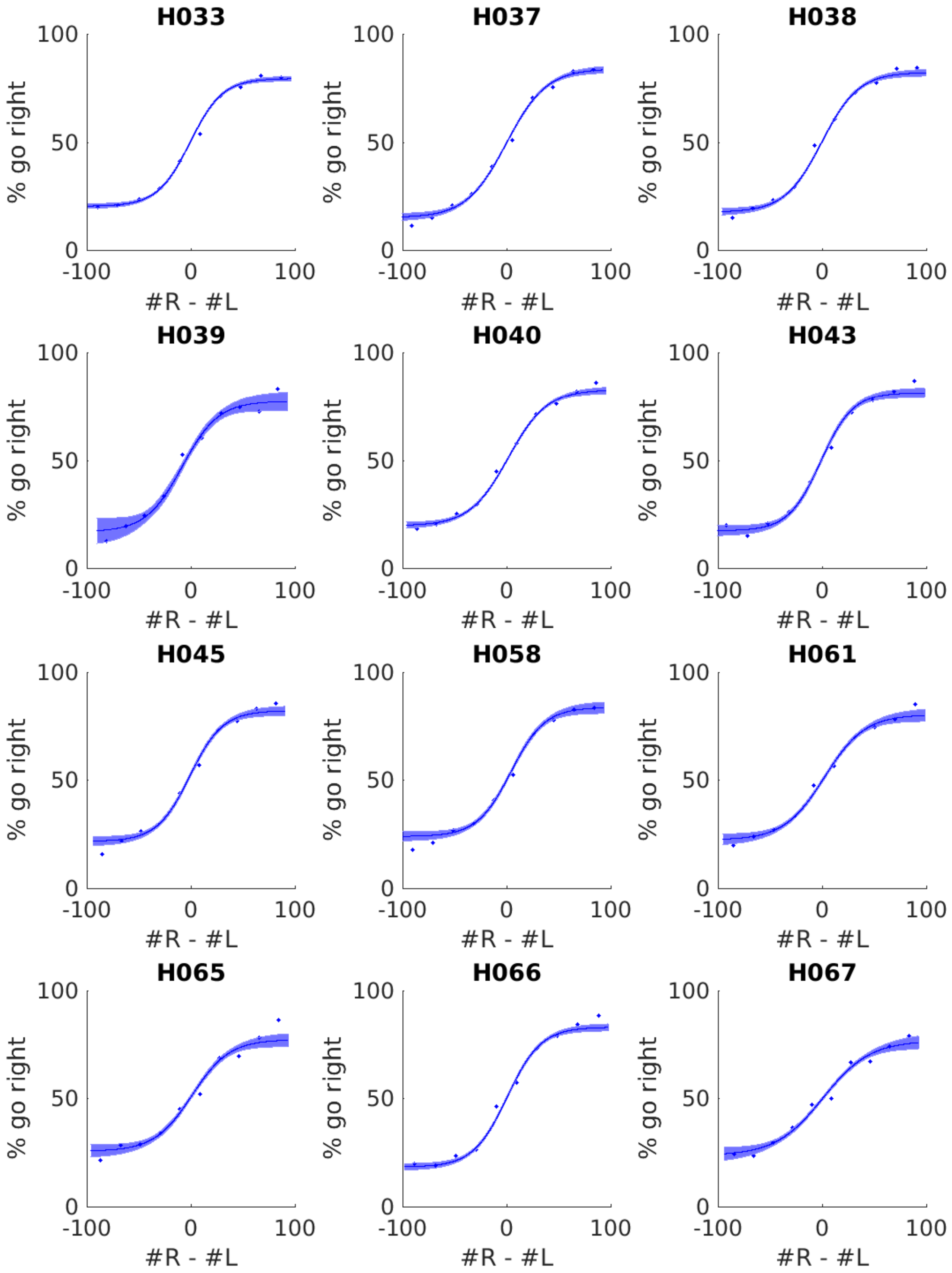

Supplementary Figure 1: **Psychometric graph for all rats** Each trial performed by the rat was binned by the total click difference in the trial. The rat's average accuracy in each bin is shown (dots). A four parameter logistic function is fit to the data with 95% confidence intervals (line).

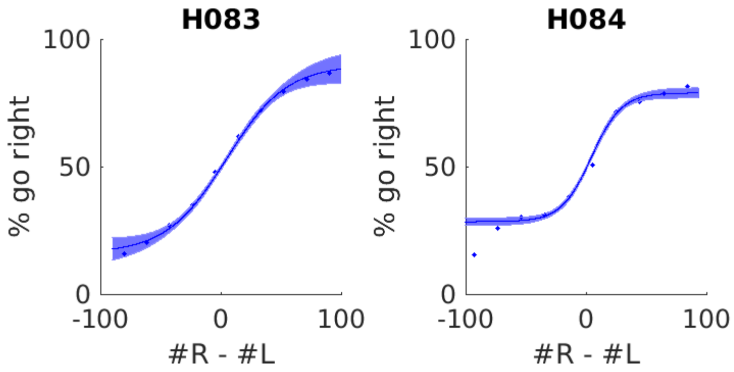

Supplementary Figure 2: **Psychometric graph for all rats** Each trial performed by the rat was binned by the total click difference in the trial. The rat's average accuracy in each bin is shown (dots). A four parameter logistic function is fit to the data with 95% confidence intervals (line).

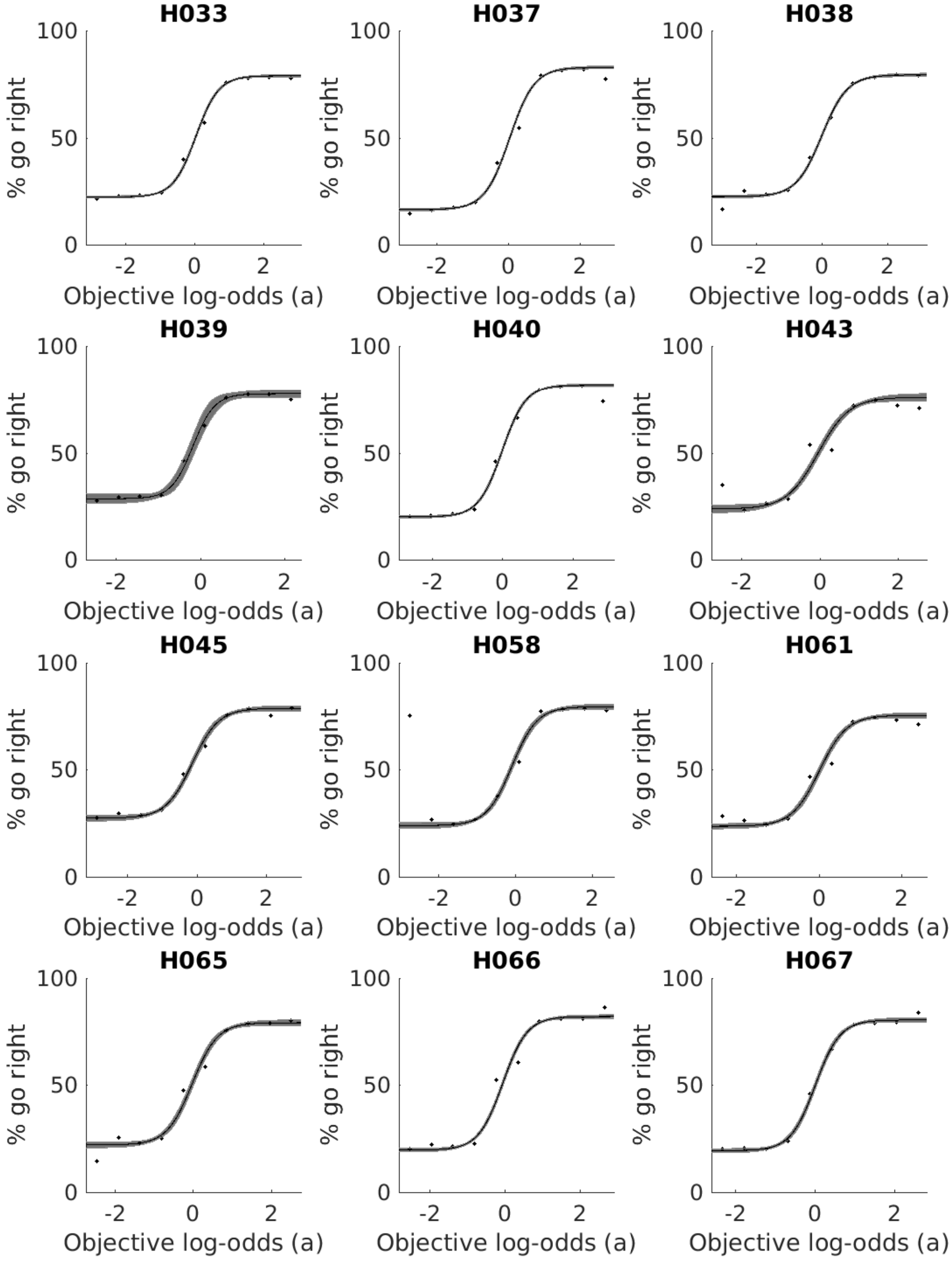

Supplementary Figure 3: **Psychometric graph for all rats against ideal observer** Each trial performed by the rat was binned by the accumulation value (log-odds) of the ideal observer (ie, no sensory noise). The rat's average accuracy in each bin is shown (dots). A four parameter logistic function is fit to the data with 95% confidence intervals (line).

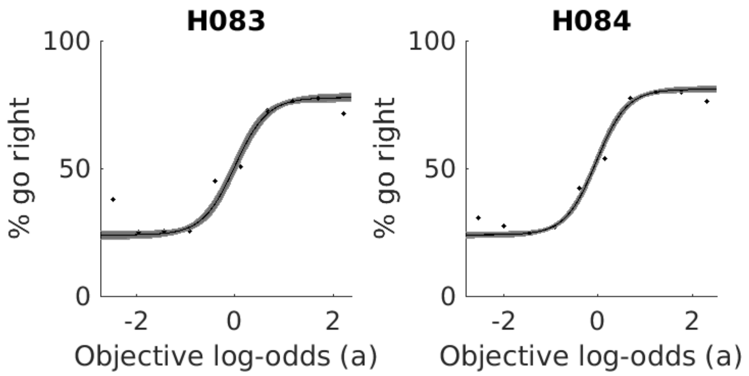

Supplementary Figure 4: **Psychometric graph for all rats against ideal observer** Each trial performed by the rat was binned by the accumulation value (log-odds) of the ideal observer (ie, no sensory noise). The rat's average accuracy in each bin is shown (dots). A four parameter logistic function is fit to the data with 95% confidence intervals (line).

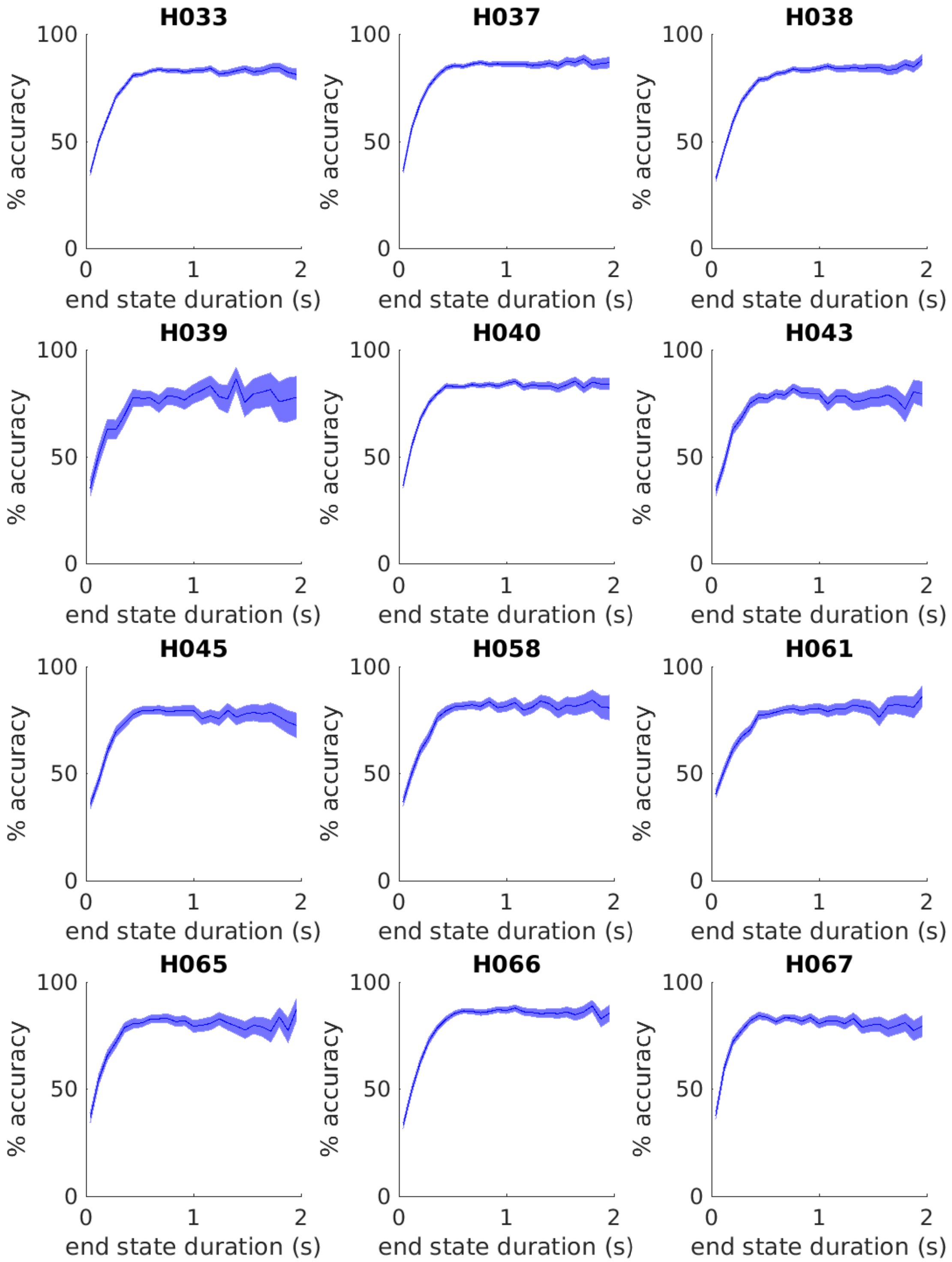

Supplementary Figure 5: **Chronometric graph for all rats with respect to final state duration** Each trial was binned by the amount of time since the last change in the hidden environmental state. The average accuracy of each bin is shown.

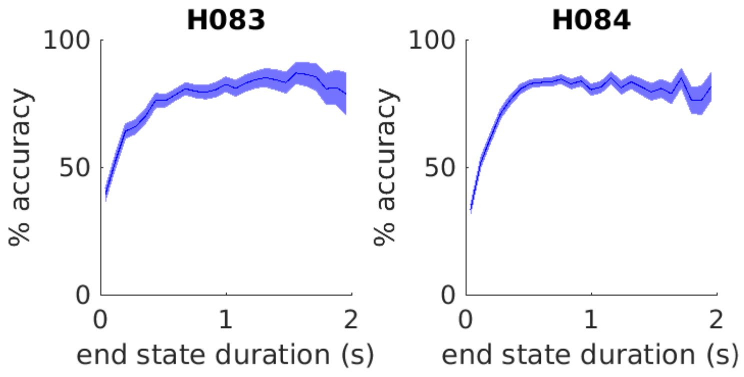

Supplementary Figure 6: **Chronometric graph for all rats with respect to final state duration** Each trial was binned by the amount of time since the last change in the hidden environmental state. The average accuracy of each bin is shown.

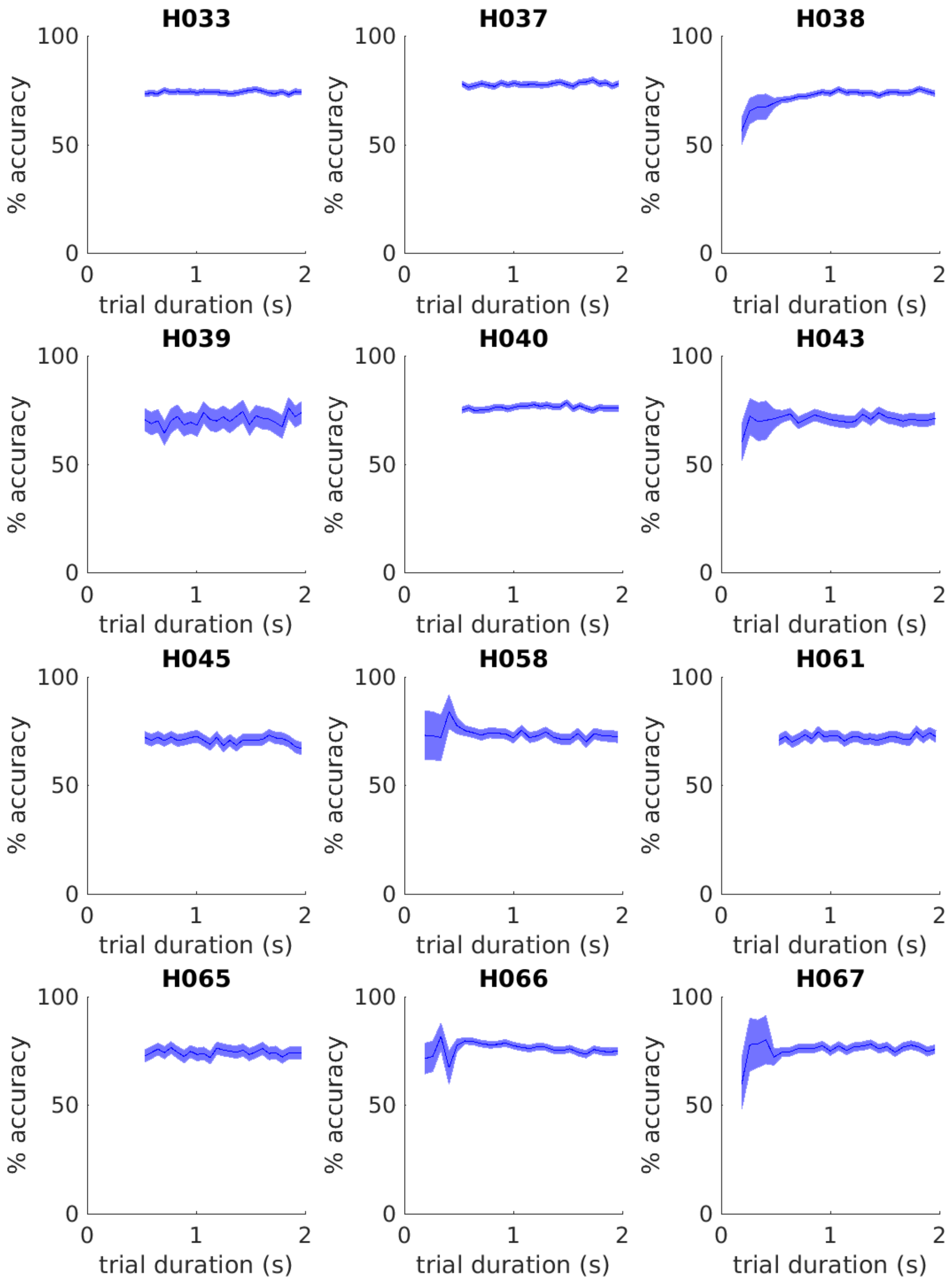

Supplementary Figure 7: **Chronometric graph for all rats with respect to total trial duration** Each trial was binned by the total trial duration. The average accuracy of each bin is shown. Most trials were drawn from the range (0.5 - 2) seconds; however, some rats experience a small number of shorter trials, leading to greater uncertainty for those durations.

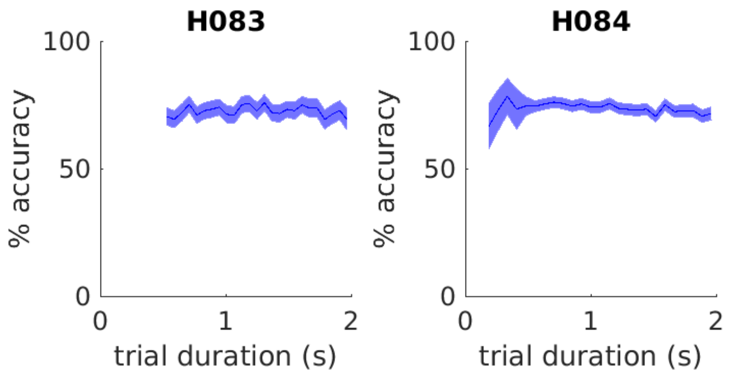

Supplementary Figure 8: **Chronometric graph for all rats with respect to total trial duration** Each trial was binned by the total trial duration. The average accuracy of each bin is shown. Most trials were drawn from the range (0.5 - 2) seconds; however, some rats experience a small number of shorter trials, leading to greater uncertainty for those durations.

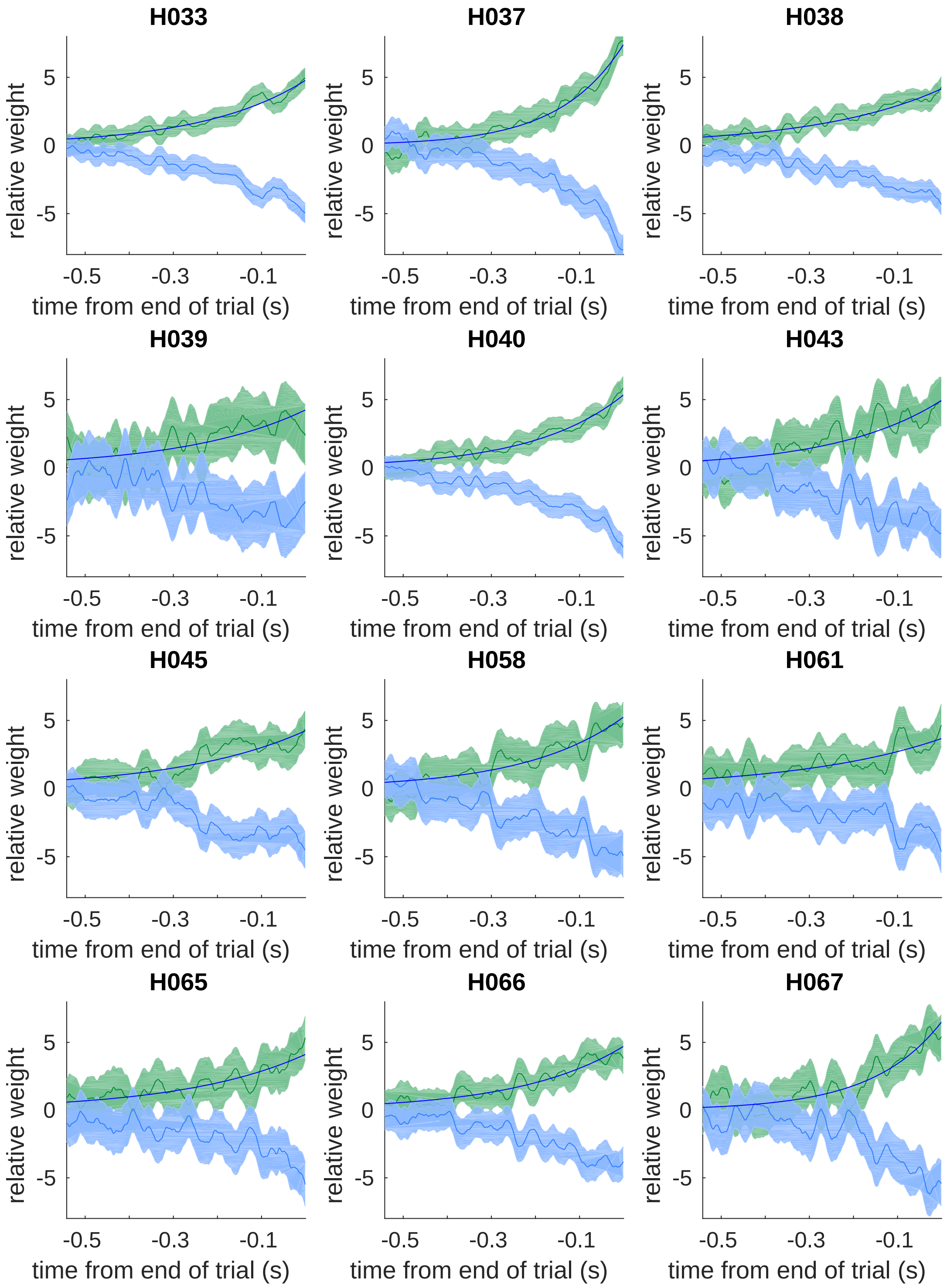

Supplementary Figure 9: **Reverse Correlation for all rats** Reverse Correlation curves for each rat (black), as well as the best fit exponential discounting function (blue).

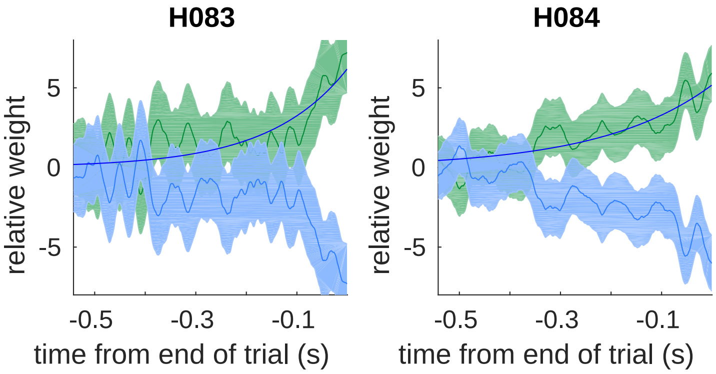

Supplementary Figure 10: **Reverse Correlation for all rats** Reverse Correlation curves for each rat (black), as well as the best fit exponential discounting function (blue).

Supplementary Note 3 - Derivation of optimal inference

Here we provide more detail on the derivation from main text equation 2 to equation 3. This derivation was developed by Ref. [3], see equations 3.2 and 3.3. However, we do not approximate the evidence term into its first two moments, instead evaluating the evidence term. For this reason we report the same derivation but halting at the intermediate step not shown in Ref. [3].

Beginning with the evidence ratio, equation 2 in the present study, and equation 3.2 in Ref. [3].

$$R_t = \frac{P(S^1|\epsilon_1 \ldots t)}{P(S^2|\epsilon_1 \ldots t)} = \frac{P(\epsilon_t|S^1)}{P(\epsilon_t|S^2)} \left(\frac{(1-h\Delta t)\, R_{t-1} + h\Delta t}{(h\Delta t)\, R_{t-1} + 1 - h\Delta t}\right). \tag{25}$$

Dividing each side by R_{t-1}

$$\frac{R_t}{R_{t-1}} = \frac{P(\epsilon_t|S^1)}{P(\epsilon_t|S^2)} \left(\frac{(1-h\Delta t)\, R_{t-1} + h\Delta t}{(h\Delta t)\, R_{t-1} + 1 - h\Delta t}\right) \frac{1}{R_{t-1}}. \tag{26}$$

Now, define $\hat{a}_t = \log(R_t)$, and take the logarithm of both sides:

$$\hat{a}_t - \hat{a}_{t-1} = \log\left(\frac{P(\epsilon_t|S^1)}{P(\epsilon_t|S^2)}\right) + \log\left(\frac{(1-h\Delta t) + h\Delta t e^{-\hat{a}_{t-1}}}{(h\Delta t)\, e^{\hat{a}_{t-1}} + 1 - h\Delta t}\right), \tag{27}$$

$$\Delta\hat{a}_t = \log\left(\frac{P(\epsilon_t|S^1)}{P(\epsilon_t|S^2)}\right) + \log\left(\frac{(1-h\Delta t) + h\Delta t e^{-\hat{a}_{t-1}}}{(1-h\Delta t) + h\Delta t e^{\hat{a}_{t-1}}}\right), \tag{28}$$

$$\Delta\hat{a}_t = \log\left(\frac{P(\epsilon_t|S^1)}{P(\epsilon_t|S^2)}\right) + \log\left(1 + h\Delta t\left(e^{-\hat{a}_{t-1}} - 1\right)\right) - \log\left(1 + h\Delta t\left(e^{\hat{a}_{t-1}} - 1\right)\right) \tag{29}$$

Using the approximation $\log(1+a) \approx a$, which is valid when $|a| << 1$. Here, $h\Delta t << 1$.

$$\Delta\hat{a}_t = \log\left(\frac{P(\epsilon_t|S^1)}{P(\epsilon_t|S^2)}\right) + h\Delta t\left(e^{-\hat{a}_{t-1}} - 1\right) - h\Delta t\left(e^{\hat{a}_{t-1}} - 1\right), \tag{30}$$

$$\Delta\hat{a}_t = \log\left(\frac{P(\epsilon_t|S^1)}{P(\epsilon_t|S^2)}\right) + h\Delta t\left(e^{-\hat{a}_{t-1}} - e^{\hat{a}_{t-1}}\right). \tag{31}$$

Using $\sinh(x) = \frac{1}{2}\left(e^x - e^{-x}\right)$:

$$\Delta\hat{a}_t = \log\left(\frac{P(\epsilon_t|S^1)}{P(\epsilon_t|S^2)}\right) - 2h\Delta t \sinh\left(\hat{a}_{t-1}\right). \tag{32}$$

Plugging in the evaluation of the log-evidence term from the main text:

$$\Delta\hat{a}_t = \left(\delta_{t,\mathrm{R}} - \delta_{t,\mathrm{L}}\right)\kappa - 2h\Delta t \sinh\left(\hat{a}_{t-1}\right). \tag{33}$$

Here, we again use $\Delta t << 1$ to justify replacing $\hat{a}_{t-1}$ with $\hat{a}_t$ on the right hand side. Evaluating the evidence term as derived in the main text, and rescaling by κ:

$$\Delta a_t = \delta_{t,\mathrm{R}} - \delta_{t,\mathrm{L}} - \frac{2h}{\kappa}\Delta t \sinh\left(\kappa a_t\right). \tag{34}$$

Taking the limit of $\Delta t \to 0$:

$$da_t = \delta_{t,\mathrm{R}} - \delta_{t,\mathrm{L}} - \frac{2h}{\kappa}\sinh\left(\kappa a_t\right) dt. \tag{35}$$

Here we are making the assumption that the action of the auditory clicks happen instantaneously with respect to the accumulation equation. Care needs to be used when interpreting the δ terms in Supplementary Equation 34 and Supplementary Equation 35. In both cases we want to each δ to increment (or decrement) the accumulation variable by a value of 1. In Supplementary Equation 34, this leads to interpreting the δ terms as Kronecker Deltas that have amplitude 1 when $R/L = t$. In Supplementary Equation 35, this leads to interpreting the δ terms as Dirac Deltas that integrate to 1 when $R/L = t$.

Supplementary Note 4 - Decreasing click reliability lengthens integration timescales

To more clearly see how less reliable clicks (smaller κ) results in a longer integration timescale, we can expand the discounting function in a taylor series around the origin:

$$f(x) \approx f(a) + \frac{f'(a)}{1!}(x-a) + \frac{f''(a)}{2!}(x-a)^2 + \frac{f'''(a)}{3!}(x-a)^3 + \ldots \tag{36}$$

$$\frac{2h}{\kappa}\sinh\left(\kappa x\right) \approx \frac{2h}{\kappa}\sinh\left(0\right) + \frac{2h}{\kappa}\frac{\kappa}{1!}\cosh\left(\kappa\cdot 0\right)(x-0) + \frac{2h}{\kappa}\frac{\kappa^2}{2!}\sinh\left(\kappa\cdot 0\right)(x-0)^2 \tag{37}$$

The even terms drop out, and we collect the odd terms:

$$\frac{2h}{\kappa}\sinh\left(\kappa x\right) \approx 2hx + \frac{2h\kappa^2}{3!}x^3 + \frac{2h\kappa^4}{5!}x^5 + \ldots \tag{38}$$

We find κ only appears with even power exponents in odd powers of x. Increasing κ will increase the strength of the discounting function. Increasing the strength of the discounting function leads to shorter integration timescales. In short, increasing κ shortens the integration timescale. Decreasing κ lengthens the integration timescale.

Supplementary Note 5 - Click mislocalization from model parameters

Our analysis of sensory noise uses an estimation of the rate of click mislocalization. Both our estimate of mislocalization for our rats, and rats from Ref. [2] are derived from parameters in the trial-by-trial model. Here we provide details on how we use those model parameters to estimate the click mislocalizaton, n.

The three model parameters that influence n are the sensory noise σ_{s}^2, the adaptation strength ϕ, and the adaptation time constant τ_ϕ. First, we compute the average level of sensory adaptation on each click, $\langle C \rangle$, by simulating the adaptation dynamics (Equation 15) on a large dataset of trials. Given the average adaptation on each click, the sensory noise σ_{s}^2 creates a Gaussian distribution of effective click amplitudes with mean $\langle C \rangle$, and variance $\sigma_{\mathrm{s}}^2 \langle C \rangle$ [2]. To compute what fraction of the probability mass would cross 0 to be registered as a click on the other side we use the Gaussian cumulative distribution function. For a standard Gaussian $\mathcal{N}\left(\mu, \sigma^2\right)$:

$$P(\text{click} \leq x) \quad = \quad \int_{-\infty}^{x} \frac{1}{\sqrt{2\pi}\sigma} e^{\frac{-(s-\mu)^2}{2\sigma^2}} ds = \frac{1}{2}\left(1 + erf\left(\frac{x-\mu}{\sqrt{2\sigma^2}}\right)\right). \tag{39}$$

Plugging in the adapted mean and variance for the average click, and $x = 0$:

$$n \quad = \quad \frac{1}{2}\left(1 + erf\left(\frac{-\langle C \rangle}{\sqrt{2\sigma_{\mathrm{s}}^2 \langle C \rangle}}\right)\right). \tag{40}$$

To estimate uncertainty on n due to uncertainty on the underlying model parameters, we performed a bootstrapping analysis. Each parameter was sampled 100 times according to the Hessian-derived covariance matrix. Each parameter sample was used to generate a sample n as described above. Figure 5C shows the maximum likelihood estimate of n and its standard error.

Supplementary Note 6 - Click mislocalization is reliable across a wide range of click rates

In order to evaluate the reliability of the click mislocalization probability, we calculated it separately for different trials in Ref. [2] based on the click rates used in the trials.

For each rat in Ref. [2], we fit all parameters of the trial-by-trial model presented in the main text using all trials performed by the rat. In this dataset, each rat performed a mixture of trials with different click rates that were randomly interleaved. Different rats performed differently sets of click rates titrated to their performance. We then fit the model separately to each subset of trials with

the same click rate. On each of these click-rate specific subsets, we fit only the three parameters that impact click mislocalization: sensory noise σ_s^2, adaptation strength ϕ, and the adaptation time constant τ_ϕ. For each model fit, we then computed the click mislocalization as defined in the previous section. Supplementary Figure 11 shows the results. The vertical pink line shows the click rates for the dynamic task, and the horizontal line shows the value of n used in our theoretical analysis. Click mislocalization is reliable across a wide range of click rates.

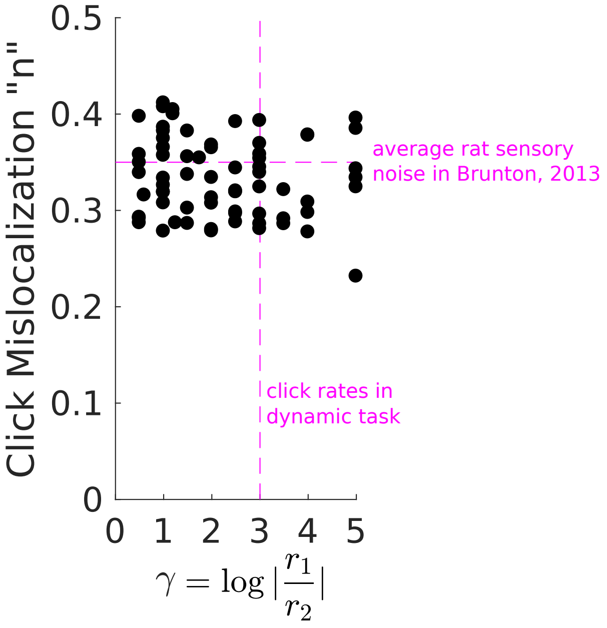

Supplementary Figure 11: **Click mislocalization across click rates is reliable.** Click mislocalization was calculated separately for trials with different click rates in Ref. [2]. Click mislocalization is constant across a wide range of click rates.

Supplementary Note 7 - Alternative sensory noise models

In this section we provide details on sensory noise. We provide analysis of different ways to parameterize sensory noise. The main analysis in the text derives optimal inference given sensory noise that is discrete, clicks are either localized on one side or the other. It is easy to imagine many other forms of sensory noise, including Gaussian fluctuations in the click amplitude, or simply missing clicks. Here we demonstrate by evaluating the log-evidence term that decreases in click reliability are primarily driven by click mislocalization, not fluctuations in the perceived amplitude of the clicks, or missed clicks. Finally, we provide a general argument for why only click mislocalization matters

Click reliability with Gaussian sensory noise

Consider Gaussian noise where the clicks played from the right/left are perceived with amplitude given by $\mathcal{N}(\pm\mu, \sigma^2)$. Here we interpret clicks with positive amplitude as right clicks, and negative amplitude as left clicks. Note that if σ^2 is sufficiently large, clicks will be mislocalized.

To compute the reliability of an individual click with a specific amplitude fluctuation, ξ, we need to compute the probability of a click generated on the right being observed with amplitude ξ: $P_{\mathrm{r}}(\xi)$, as well as the probability of a click generated on the left being observed with amplitude ξ: $P_{\mathrm{l}}(\xi)$. Formally we need to integrate the Gaussian probability density function over a small window centered at ξ.

$$P_{\mathrm{r}}(\xi) = \int_{\xi-\epsilon}^{\xi+\epsilon} \frac{1}{\sqrt{2\pi\sigma^2}} e^{-\frac{(\mu-s)^2}{2\sigma^2}} ds \tag{41}$$

$$P_{\mathrm{l}}(\xi) = \int_{\xi-\epsilon}^{\xi+\epsilon} \frac{1}{\sqrt{2\pi\sigma^2}} e^{-\frac{(-\mu-s)^2}{2\sigma^2}} ds \tag{42}$$

$$\kappa\left(r_1, r_2, P_{\mathrm{r}}, P_{\mathrm{l}}\right) = \log \frac{(r_1\Delta t)P_{\mathrm{r}}(1-r_2\Delta t) + (1-r_1\Delta t)(r_2\Delta t)P_{\mathrm{l}}}{(r_2\Delta t)P_{\mathrm{r}}(1-r_1\Delta t) + (1-r_2\Delta t)(r_1\Delta t)P_{\mathrm{l}}}. \tag{43}$$

This expression for κ seems hard to interpret, but notice what happens if $P_{\mathrm{l}} = 0$.

$$\kappa\left(r_1, r_2, P_{\mathrm{r}}, 0\right) = \log \frac{(r_1\Delta t)(1-r_2\Delta t)}{(r_2\Delta t)(1-r_1\Delta t)} = \kappa(r_1, r_2). \tag{44}$$

In this case, P_{r} drops out entirely, and we get the same value of κ as the no-noise case. This demonstrates that click mislocalization is necessary for a decrease in click reliability.

Next, we will compare how the Gaussian click reliability scales with the rate of mislocalization. We generated a dataset of trials where each click had an amplitude drawn from a Gaussian distribution. We asked what was the accuracy of the nonlinear inference using the Gaussian click reliability derived above,

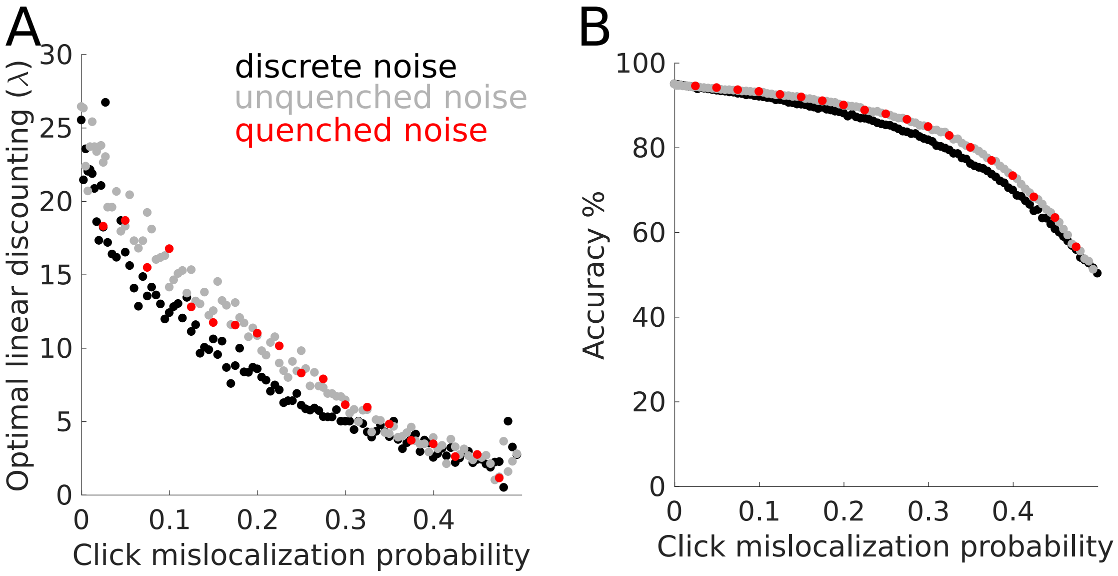

Supplementary Figure 12: **Three Interpretations of Gaussian noise.** (A) Numerically optimized linear discounting rates for different noise amplitudes. (B) Accuracy from the numerical optimized linear discounting rates shown in panel A. (A and B) (Black dots) Discrete noise, same points as Figure 2. (Grey dots) unquenched Gaussian noise of the form in Supplementary Equation 46. The unqueched fluctuations favor a larger discounting rate.

and what is the discounting rate of the best linear discounting agent? We refer to this as "quenched Gaussian noise," the meaning of quenched is explained below. We then considered a second dataset where the Gaussian amplitudes were thresholded to either be ± 1 reflecting whether the amplitude was above or below 0. We refer to this as "discrete noise." We compute the click mislocalization probability for corresponding to each Gaussian variance σ^2 by:

$$\langle n(\mu, \sigma^2) \rangle \quad = \frac{1}{2}\left(\mu + erf\left(\frac{1}{\sqrt{2\sigma_s^2}}\right)\right) \tag{45}$$

Supplementary Figure 12 shows the results of the comparison. The discrete noise has a slight decrease in accuracy, and a slightly smaller discounting rate. The difference is due to clicks that weakly change sign. The discrete noise doesn't distinguish between small and large amplitude clicks, where the quenched Gaussian noise does. Importantly, in the noise regime we expect the rats, there is no difference between these interpretations of sensory noise.

Unquenched Gaussian noise in the trial-by-trial model

Gaussian noise subjects the clicks to large amplitude fluctuations in how they are perceived. Our trial-by-trial model handles these fluctuations slightly differently from the normative theory outlined in the section above. First, observe that in the optimal inference theory, the evidence reliability term quenches large amplitude fluctuations. Following the derivation in the section above, $\kappa\left(r_1, r_2, P_{\rm r}, P_{\rm l}\right)$

is bounded between $\pm\kappa\,(r_1, r_2)$, so the evidence added to the accumulation variable after each click is bounded ("quenched") and not subjected to large amplitude fluctuations.

Second, we asked whether the presence of large amplitude fluctuations of click amplitudes if they are not quenched, would cause a linear approximation to favor a stronger evidence discounting in order to damper the fluctuations. Specifically, we asked whether an evidence discounting agent with unquenched Gaussian noise:

$$da = (\delta_{\mathrm{R},t} - \delta_{\mathrm{L},t})\,\mathcal{N}(1, \sigma^2) - \lambda a dt, \tag{46}$$

would maximize accuracy with a larger λ than the same click mislocalization strength implemented as quenched noise in the normative theory. Quenched noise as properly implemented in the normative theory would look like:

$$da = (\delta_{\mathrm{R},t} - \delta_{\mathrm{L},t})\,\kappa\left(r_1, r_2, \mathcal{N}(1, \sigma^2)\right) - \lambda a dt. \tag{47}$$

Supplementary Figure 12 shows a comparison between quenched and unquenched Gaussian noise. We find no difference between these interpretations. In panel B, the accuracy of the unquenched Gaussian noise is from the best linear discounting agent, because we do not have a normative theory for unquenched noise (precisely what the simulation was asking to compare).

Click reliability with missed clicks

An alternative form of sensory noise might parameterize the probability that a subject just fails to hear a click at all. Using this framework, we show that missed clicks doesn't change the click reliability function. Assume a click that is generated is not detected at all with probability m. Then, the click reliability of a click on the right can be computed as:

$$\kappa(r_1, r_2, m) = \log\left(\frac{r_1(1-m)r_2 m + r_1(1-m)(1-r_2)}{r_2(1-m)r_1 m + r_2(1-m)(1-r_1)}\right) \tag{48}$$

We can interpret this expression as the probability of having a click be generated on one side and not missed and a click generated on the other side and missed, or the probability of a click being generated on one side and not being missed and no click is generated on the other side. Given that $\Delta t << 1$, we can remove second order terms in Δt:

$$\kappa(r_1, r_2, m) = \log\left(\frac{r_1(1-m)}{r_2(1-m)}\right) = \log\left(\frac{r_1}{r_2}\right) \tag{49}$$

We find the click reliability is independent of the probability of missing a click, m.

A general argument for click mislocalization

In the previous sections we demonstrated that in the case of missed clicks, or gaussian clicks, mislocalization is necessary for decreasing click reliability. Here we provide a general argument for why that is true under any form of sensory noise. The auditory evidence takes on two possible values $S = \{+1, -1\}$. Let y be the value of each auditory stimuli after being noisily encoded by the sensory transduction process ($y = f(S)$). If $f()$ maps left and right clicks separately into non-overlapping distributions of click amplitudes, then an ideal observer can bin y into groups $y < 0$ and $y > 0$, and perfectly recover the original signal S. If $f()$ maps left and right clicks into overlapping distributions, then an observer cannot bin y to perfectly recover the original signal. If the observer uses the same binary binning scheme as before, then the error rate in the recovered signal will be equal to the mislocalization rate. Notice that an observer with perfect knowledge of the distribution of $f()$ can do slightly better by using a different binning scheme. If the observer recognizes that clicks in the domain where the left and right distributions overlap are less trustworthy, then the observer can use multiple bins to discount specifically those clicks near 0. The Gaussian reliability function above $\kappa(r_1, r_2, P_\mathrm{r}, P_\mathrm{l})$ can be considered an observer with an infinite number of bins. As seen in Supplementary Figure 12, this strategy slightly improves accuracy above the two-binning strategy. We thus conclude that click mislocalization is the source of decreasing click reliability from sensory noise.

Supplementary Note 8 - Linear approximation details

We performed three analyses in order to demonstrate that the linear approximation is difficult to distinguish from the full nonlinear inference process on a trial-by-trial basis. First, we compared the cross-consistency between models to the self-consistency of each model across repeated simulation of the same trials with different sensory noise realizations. The nonlinear inference process, and the linear approximation were simulated on a dataset of 30,000 trials. The simulations were run 20 times with different noise realizations on each simulation. We calculated on average how self-consistent the linear (71.98% $\pm$ 0.48) and nonlinear (72.87% $\pm$ 0.52) models were across noise realization. In addition, we calculated the cross-consistency between the linear and nonlinear models when the noise realization was different (72.40% $\pm$ 0.50), or the same (96.98% $\pm$ 0.18). The value reported is the average percentage agreement between all possible pairs in each group with 95% confidence intervals. Given that the cross-consistency between models is the same as the self-consistency for each model, we conclude the two models are hard to distinguish, and have similar predictive ability on new trials. Importantly, the linear model achieves similar behavior with one less parameter.

Our second analysis looked at the small set (3%) of trials where the nonlinear and linear models disagreed when given the same noise realization. Trial difficulty on our task is determined by the length of time since the last hidden state switch. We found that the two models disagreed slightly more often immediately after a state transition, but overall disagreements were spread across all trials (Supplementary Figure 13C). We then computed the chronometric curves which show model accuracy as a function of time since the last hidden state switch. We find the two models have overlapping confidence intervals at all time points (Supplementary Figure 13B). We conclude it would be difficult to distinguish these two models on this dataset.

Finally, to provide intuition as to how a linear function could accurately approximate a nonlinear function we computed the distribution of values the nonlinear model would produce after being simulated on a large dataset of trials (Supplementary Figure 14). The discounting term bounds how much evidence can accumulate for either of the two states. This creates a distribution of evidence values over a narrow domain. Comparing this distribution with the linear and nonlinear discounting functions, we see the linear approximate is close to the nonlinear function in the same domain as the distribution of accumulation values. This provides intuition as to how the linear function is able to approximate the nonlinear discounting function.

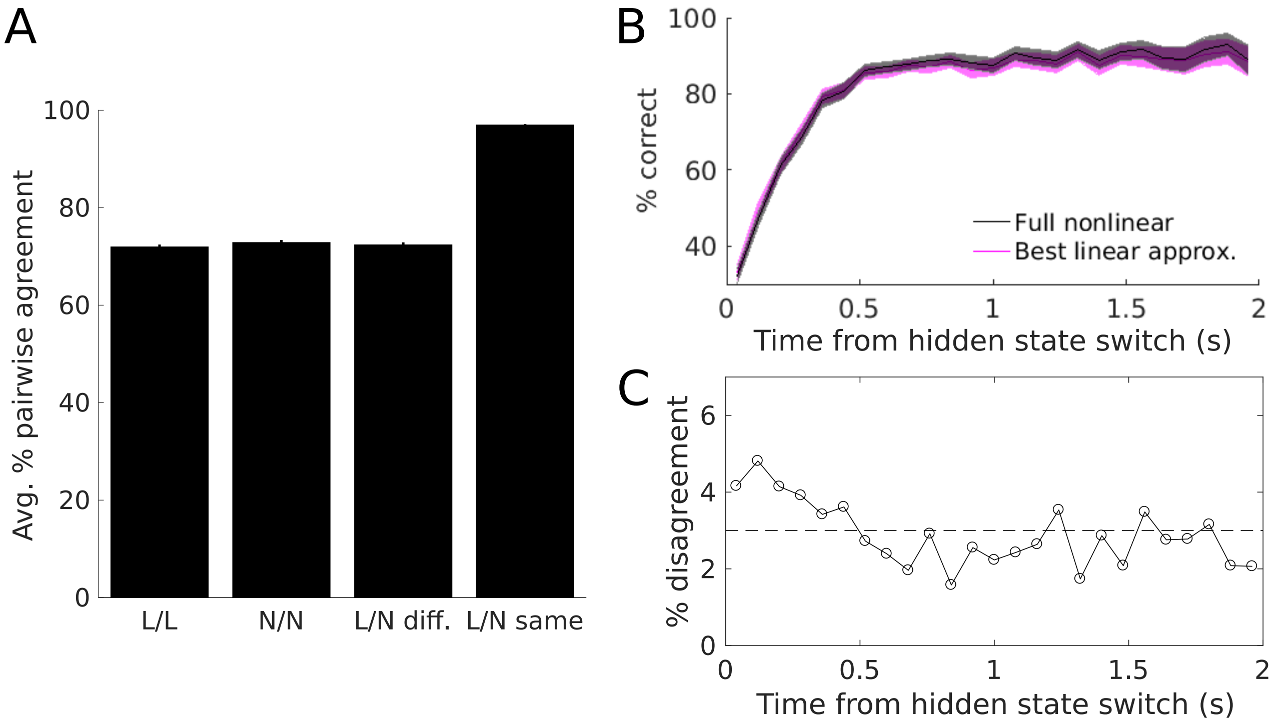

Supplementary Figure 13: **Linear approximation is hard to distinguish from the full nonlinear inference.** (A) Average pairwise agreement across repeated simulations of the same trials with different noise realizations for the linear (L) and nonlinear (N) models. Panel shows the self consistency for the linear (L/L) and nonlinear (N/N) models, as well as the cross-consistency with the same noise (L/N same), and different noise (L/N diff.). (B) Chronometric plot shows accuracy for both models as a function of time since the last hidden state switch, simulated on a dataset of 30,000 trials with the same noise realization. Shaded bands show 95% confidence intervals. The two models have the same pattern of accuracy over time. (C) Same simulations as in B, but showing the percentage of trials where the two models disagree over time. The dotted line shows the overall percentage of trials where the models disagree. The models disagree slightly more often immediately following state changes, but consistently disagree at the same rate across time.

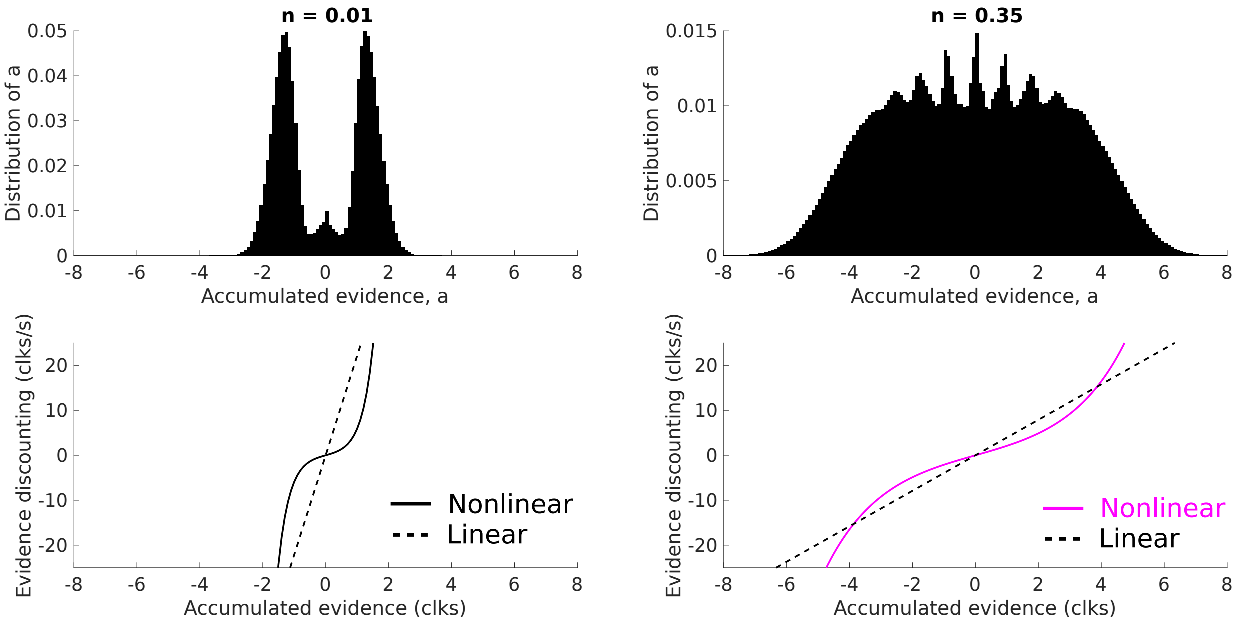

Supplementary Figure 14: **Linear approximation is accurate in the domain of distribution of accumulation values** To provide intuition as to how a linear function could accurately approximate the highly nonlinear discounting function we compare the distribution of accumulation values to the domain of the discounting functions. (Top) the distribution of accumulation value, a, from simulating the full nonlinear discounting theory on a dataset of trials. (Bottom) The full nonlinear and best linear approximation of the discounting functions. The discounting function bounds how far away from zero the evidence can accumulate. The linear approximation is close to the nonlinear function in precisely the same domain as the distribution of accumulated evidence.

Supplementary Note 9 - Psychophysical reverse correlation details

Here we present four control analyses on our reverse correlation method. First, we show our reverse correlation derived time constants with confidence intervals. Main figure text omits them for readability. Second, we show that our method is not biased by the presence of a lapse rate, unlike logistic regression. Third, we rule out degenerate strategies like deciding based on only the last click. Fourth, we rule out an alternative hypothesis to explain the observed discounting rates based on poor estimation of the environmental volatility.

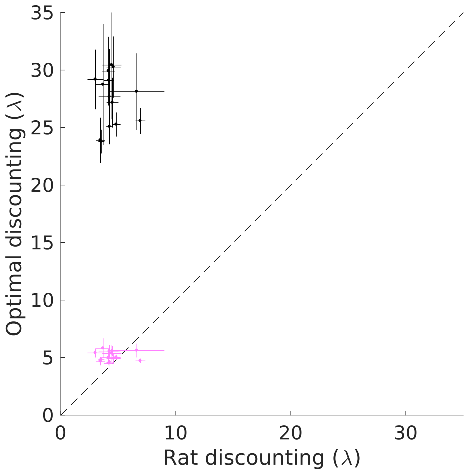

Supplementary Figure 15: **Reverse Correlation Timescales with uncertainty**. Same analysis as Figure 4B, but with 95% confidence intervals on the exponential time constant fits.

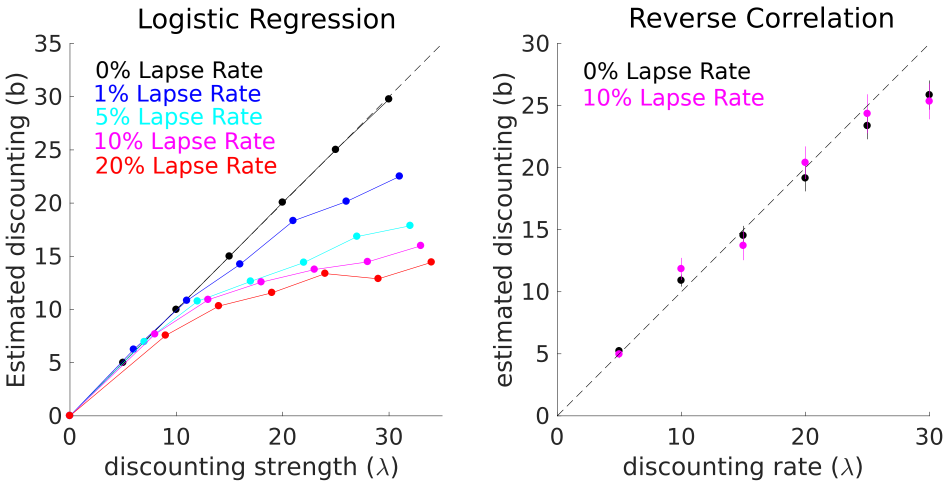

Supplementary Figure 16: **Reverse Correlation timescales are unaffected by lapse rates**. Lapse rates are defined as the percentage of trials where the subject makes a random response. (Left) Logistic regression is strongly biased by the presence of a lapse rate. (Right) Psychophysical reverse correlation methods used in this study are not biased.

Supplementary Note 10 - Ruling out last click strategies

One possible concern is that the rats might be relying on degenerate strategies like choosing based on the last click they heard. Or that the rat's integration timescale is so short, that their behavior shouldn't really be considering integration. Supplementary Figure 17A shows a quasi-fixed point analysis of the optimal accumulation equation given a noise level. Assuming the environment stays in one state for a long time, we then replace the evidence term with the expected rate of clicks, and solve for the steady state accumulation value. We can see that for all noise levels, the fixed point lies above 1 click, so the optimal behavior necessarily involves integrating clicks. For the average rat noise level, we see integration of about 5 clicks. We conclude that the ideal strategy involves integrating many clicks.

Supplementary Figure 17B shows the recovered discounting rate from the reverse correlation method against a simulated discounting agents, similar to Figure 3. Here, we include much stronger discounting agents, and find the recovered discounting rate asymptotes at just under 36 Hz, which is the expected total click rate($r_1 - r_2 \approx 36$). The last click strategy could be considered a discounting agent with an infinite discounting rate, and would be recovered in our analysis as a discounting rate of about 36. We find our rats are well away from this limit. Thus we confidently rule out a last click strategy.

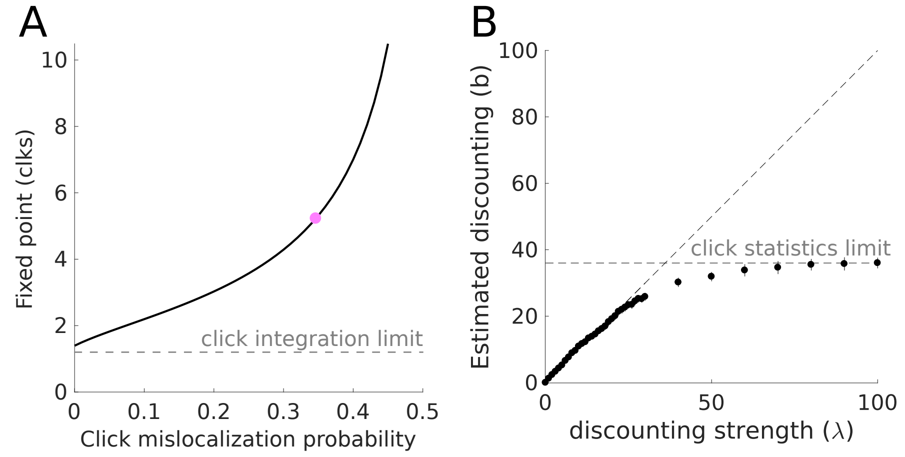

Supplementary Figure 17: **Ruling out last click strategies.** (A) Quasi fixed points derived from the expected click rate and evidence discounting functions, assuming a fixed environmental state. For all noise levels, the fixed point is greater than 1 click. (B) Integration timescales measured from reverse correlation curves. At large discounting rates, the timescale saturates reflecting the timescale of click generation.

Supplementary Note 11 - Ruling out poor estimates of environmental volatility

Ref. [4] examined human discounting behavior on a comparable task, finding that human subjects failed to discount at the optimal rate due to poor estimates of the environmental volatility. Our linear approximation to the full inference process does not directly parameterize environmental volatility. In order to evaluate whether our rat's discounting rates could be explained by a poor estimate of environmental volatility rather than sensory noise, we trained two rats on trials with $r_1 = 40hz$, and $r_2 = 0hz$. If the rats had no sensory noise, then the reliability of each click at informing the current state becomes infinite $\kappa(r_1, r_2) = \log\left(\frac{r_1}{r_2}\right) = \infty$. An infinite click reliability means the rats could achieve perfect accuracy by choosing the side based on the last click played. Crucially, with an infinite click reliability, the estimate of the environmental volatility becomes irrelevant. If the observed discounting rates in the main text were driven by poor estimates of the environmental volatility and not sensory noise, then in this high reliability environment the rats would make their choices based on the last click. However, if sensory noise is present, then the theory presented in the main text predicts the rats should discounting evidence at a rate comparable to the main results. Supplementary Figure 18A shows the reverse correlation discounting rates, comparable to Figure 4B. In addition, the discounting parameter for the model fit to these rats is displayed. We see the rats do not adopt a last-click strategy, consistent with sensory noise being the primary driver of the rat's discounting rates. Supplementary Figure 18B shows the session by session accuracy for each rat, showing no trend of increasing accuracy or adaptation to the high click reliability environment.

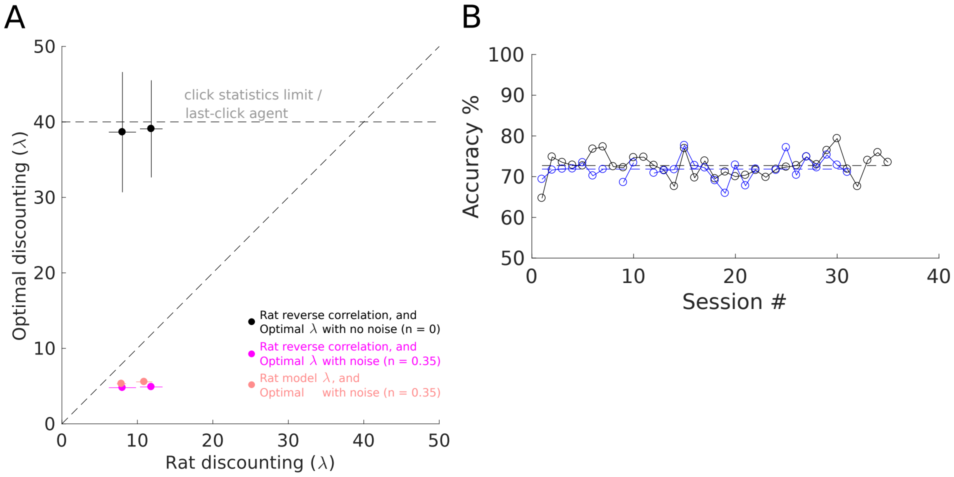

Supplementary Figure 18: **Rats in a high click reliability environment behave consistently with high sensory noise, not poor volatility estimation.** (A) Quantification of discounting timescales for rats in a high reliability environment. Reverse correlation integration timescales for rats in a high reliability environment ($r_1 = 40$, $r_2 = 0$), compared to optimal linear models with and without sensory noise. Model discounting parameters are also plotted. The horizontal dashed line shows the discounting rate expected from a last-click agent. (B) Accuracy for each behavioral session for two rats. Dotted line is average accuracy across all sessions. No apparent trend in increasing accuracy to suggest the rats are adapting to the high click reliability environment.

Supplementary Note 12 - Trial-by-trial model details

In this section we provide additional analysis on our trial-by-trial model. First, we provide parameter estimates and parameter uncertainty values for each rat, and compare them to rats from Ref. [2] (Supplementary Figure 19, Supplementary Table 1). Second, we plot model residual error against time (Supplementary Figure 20/21). Third, we show an analysis which rules out significant parameter trade-offs involving the evidence discounting parameter λ (Supplementary Figure 22).

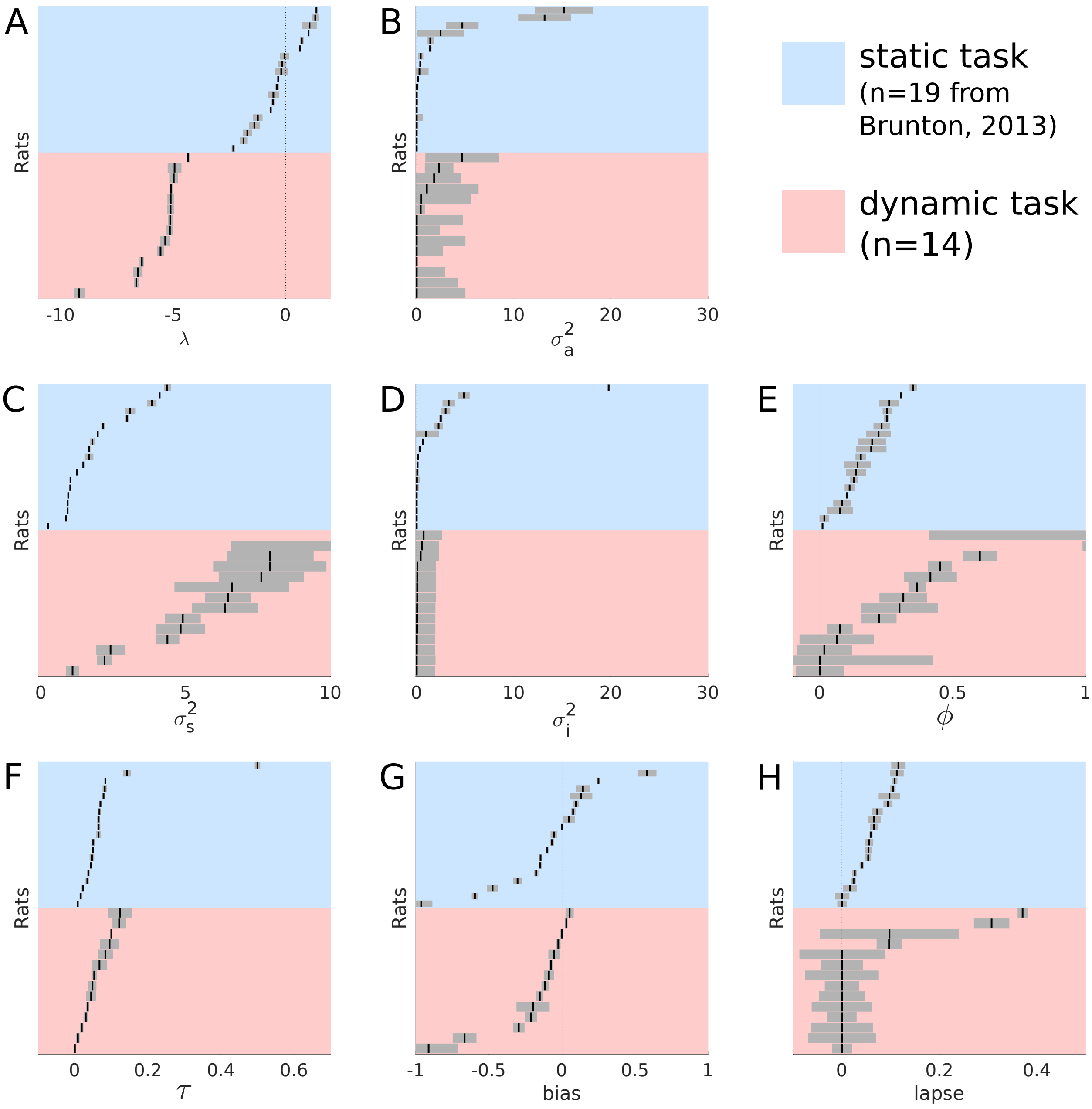

Supplementary Figure 19: **Best fitting model parameters on static and dynamic tasks.** The best fitting parameters and their standard errors are shown for each rat in the current study, compared to each rat from Ref. [2]. Each parameter plot has the rats sorted independently by parameter value, rows across panels do not indicate the same rat. (A) Evidence discounting parameter λ. (B) Accumulation noise variance. (C) Sensory noise variance. (D) Initial noise variance. (E) Adaptation Strength. (F) Adaptation time constant. (G) Decision bias parameter. (H) Lapse rate.

rat	λ	σ_a^2	σ_s^2	σ_i^2	ϕ	τ_ϕ	Bias	Lapse	λ_{opt}
H033	-5.09 ± 0.07	1.79 ± 2.76	6.45 ± 0.79	0.060 ± 1.87	0.45 ± 0.045	0.053 ± 0.0073	-0.025 ± 0.014	$1.1e-6 \pm 0.042$	-5.03 ± 0.014
H037	-6.39 ± 0.092	0.41 ± 0.44	2.19 ± 0.26	0.0076 ± 1.87	0.37 ± 0.032	0.12 ± 0.018	0.030 ± 0.0072	$3.6e-9 \pm 0.020$	-5.92 ± 0.0079
H038	-4.33 ± 0.063	$2e-4 \pm 4.99$	11.6 ± 0.99	0.71 ± 1.86	1.20 ± 0.79	$1.2e-4 \pm 1.7e-4$	-0.21 ± 0.039	0.097 ± 0.025	-5.13 ± 0.091
H039	-4.94 ± 0.30	0.44 ± 5.11	11.52 ± 4.96	0.052 ± 1.87	$5.3e-4 \pm 0.42$	0.0083 ± 0.0042	-0.91 ± 0.20	0.097 ± 0.14	-4.56 ± 0.13
H040	-6.64 ± 0.099	2.30 ± 1.45	4.36 ± 0.40	0.072 ± 1.87	0.075 ± 0.047	0.035 ± 0.0034	-0.073 ± 0.012	$1.9e-7 \pm 0.029$	-5.53 ± 0.079
H043	-4.98 ± 0.19	1.04 ± 5.3	7.61 ± 1.47	0.052 ± 1.87	1.02 ± 0.029	0.12 ± 0.032	-0.20 ± 0.11	0.31 ± 0.036	-5.94 ± 0.0022
H045	-5.11 ± 0.13	0.0029 ± 2.37	1.09 ± 0.23	0.028 ± 1.87	$4.8e-4 \pm 0.089$	0.048 ± 0.010	-0.67 ± 0.079	0.37 ± 0.0098	-5.27 ± 0.18
H058	-5.15 ± 0.15	$3.59e-5 \pm 2.92$	4.82 ± 0.84	$6.64e-5 \pm 1.87$	0.42 ± 0.098	0.10 ± 0	-0.15 ± 0.023	$1.64e-7 \pm 0.063$	-4.87 ± 0.42
H061	-5.12 ± 0.15	4.69 ± 3.78	6.59 ± 1.97	0.038 ± 1.87	0.31 ± 0.089	0.067 ± 0.019	0.053 ± 0.026	$1e-6 \pm 0.087$	-4.75 ± 0.0026
H065	-6.57 ± 0.20	$1.53e-5 \pm 4.99$	7.91 ± 1.49	0.0065 ± 1.87	0.064 ± 0.14	0.019 ± 0.0040	-0.089 ± 0.033	$4.09e-8 \pm 0.069$	-5.37 ± 0.0054
H066	-5.13 ± 0.089	$5.9e-5 \pm 2.68$	4.89 ± 0.61	0.52 ± 1.71	0.60 ± 0.063	0.084 ± 0.019	-0.12 ± 0.022	$3.8e-7 \pm 0.035$	-5.58 ± 0.27
H067	-9.18 ± 0.23	$3.9e-5 \pm 0$	2.40 ± 0.49	0.40 ± 1.84	0.22 ± 0.065	0.095 ± 0.026	-0.0012 ± 0.077	$3.3e-7 \pm 0.047$	-6.05 ± 0.081
H083	-5.35 ± 0.22	0.0034 ± 4.75	7.90 ± 1.95	0.028 ± 1.87	0.30 ± 0.14	0.045 ± 0.013	-0.053 ± 0.038	$9.5e-7 \pm 0.075$	-4.87 ± 0.025
H084	-5.56 ± 0.014	$3.5e-5 \pm 4.21$	6.35 ± 1.12	$1.02e-4 \pm 1.91$	0.017 ± 0.103	0.029 ± 0.0054	-0.29 ± 0.037	$2.3e-7 \pm 0.062$	-5.17 ± 0.12

Supplementary Table 1: **Maximum likelihood parameters and the standard error for each parameter.**

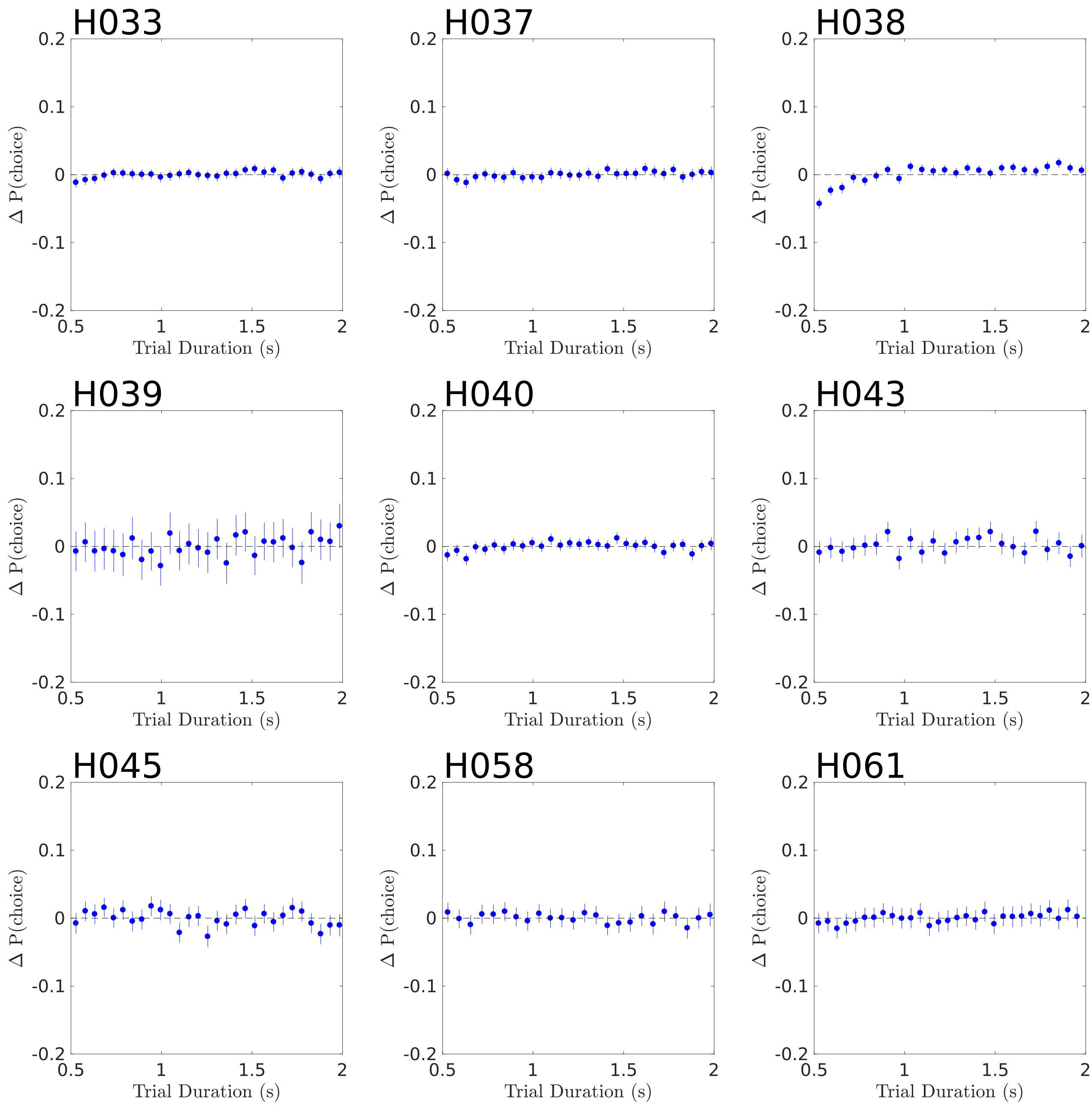

Supplementary Figure 20: **Model Residual error against time** The model fits short and long trials equally well.

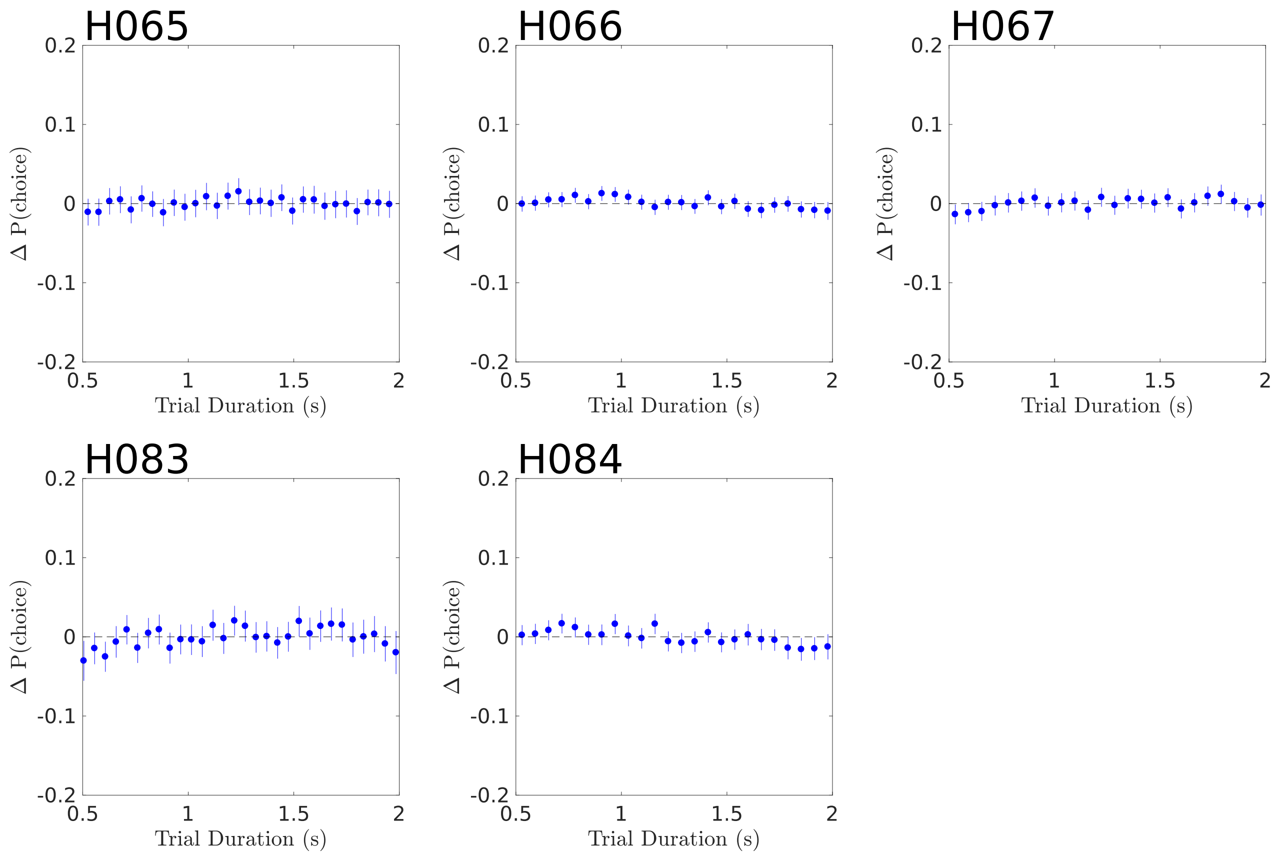

Supplementary Figure 21: **Model Residual error against time** The model fits short and long trials equally well.

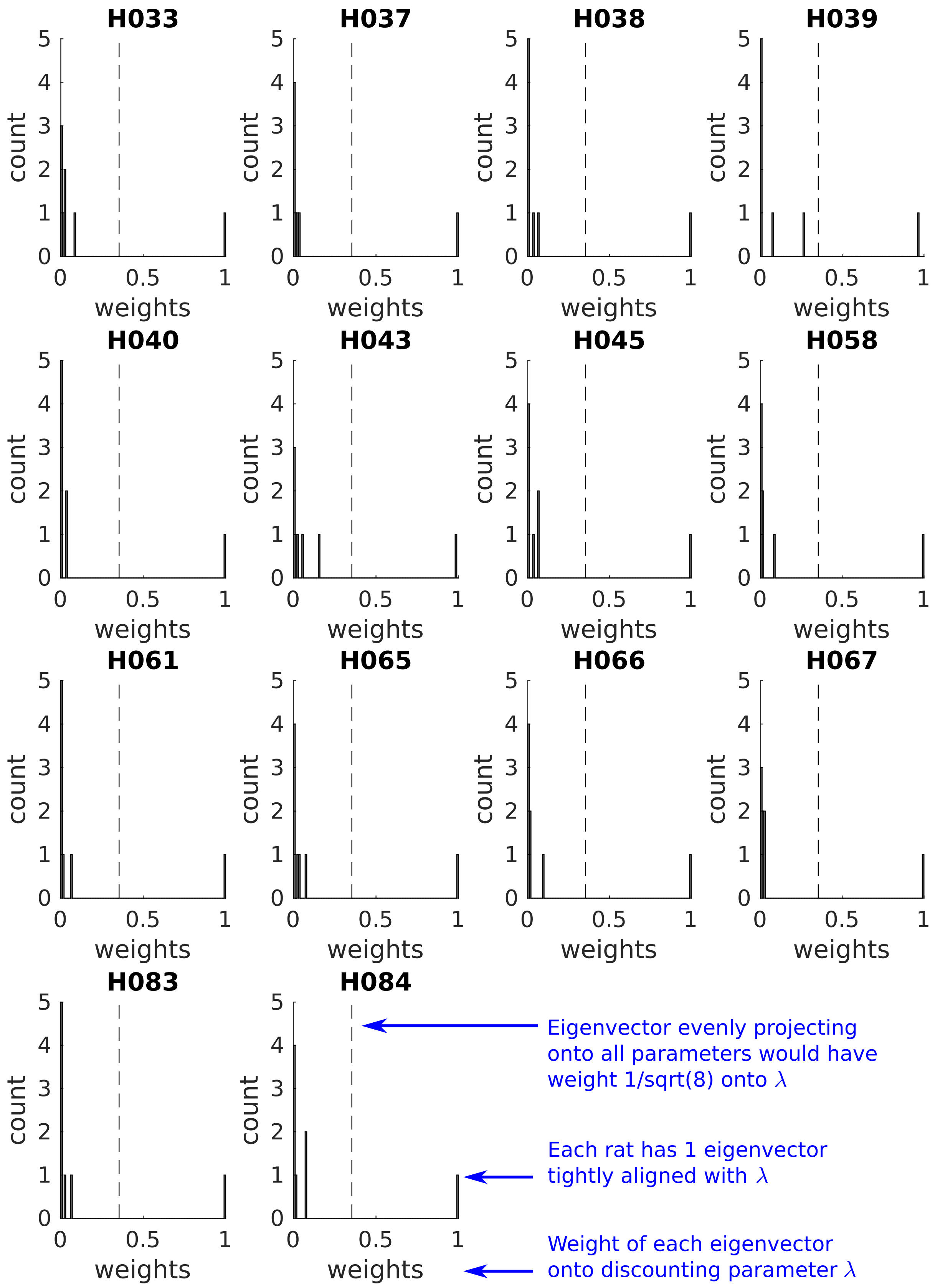

Supplementary Figure 22: **No parameter trade-offs involving evidence discounting λ.** Eigenvector decomposition of Hessian approximation of likelihood landscape at best fit parameters demonstrates no significant parameter trade-offs involving the evidence discounting parameter λ. For each rat, a histogram of eigenvector weights on λ. Each rat has one eigenvector tightly aligned with λ. The vertical dashed line indicates the weight that would result from an eigenvector that projected equally on to all parameters. Parameter trade-offs would appear as an eigenvector with weight greater than the dashed line, and less than one.

Supplementary Note 13 - Individual Rats in different environments

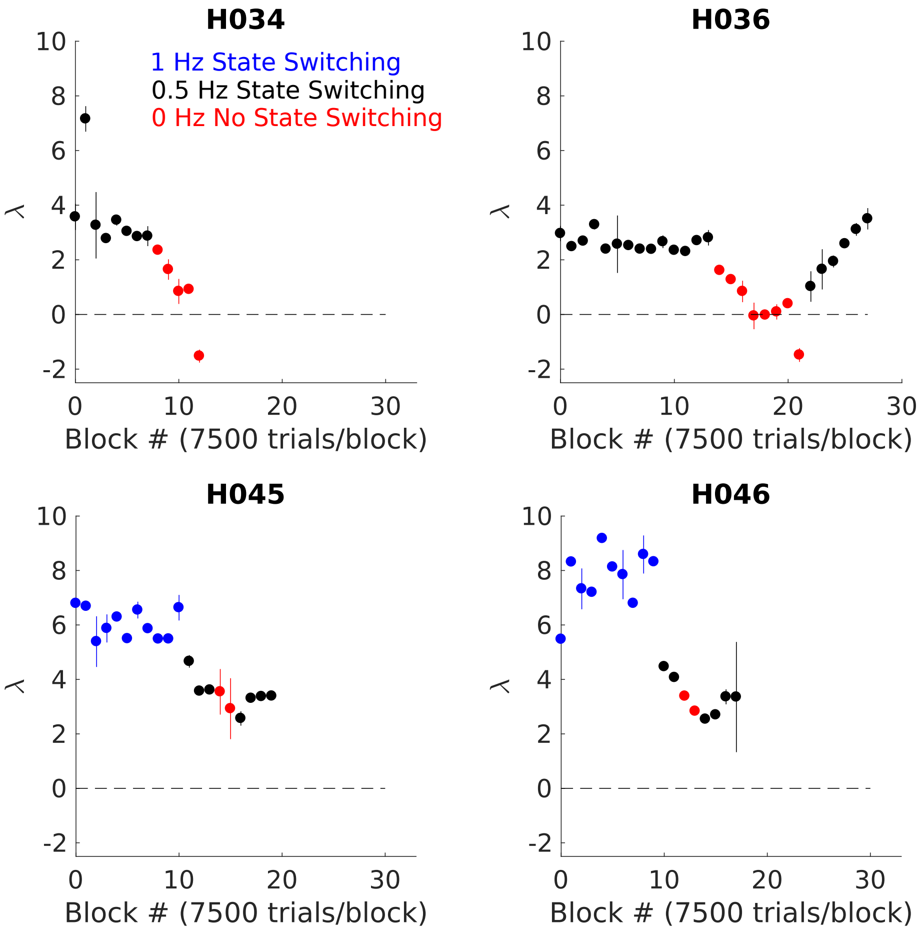

Supplementary Figure 23: **Discounting Rates change in response to environmental volatility** The discounting parameter for each rat in blocks of 7500 trials over changing environmental statistics. Each rat was moved from a hazard rate = 0.5Hz environment, to a static environment, and back to a 0.5 hz environment. Rat H034 was removed from training during the 0Hz block and thus did not perform the return to 0.5 hz trials. Rats H045 and H046 were first moved from a 1Hz environment to a 0.5 Hz environment before moving to a static environment.

Supplementary References

[1] Duan, C. A., Erlich, J. C. & Brody, C. D. Requirement of prefrontal and midbrain regions for rapid executive control of behavior in the rat. *Neuron* **86**, 1491 – 1503 (2015).

[2] Brunton, B. W., Botvinick, M. M. & Brody, C. D. Rats and humans can optimally accumulate evidence for decision-making. *Science* **340**, 95–98 (2013).

[3] Veliz-Cuba, A., Kilpatrick, Z. P. & Josic, K. Stochastic models of evidence accumulation in changing environments. *SIAM Review* (2016).

[4] Glaze, C. M., Kable, J. W. & Gold, J. I. Normative evidence accumulation in unpredictable environments. *eLife* **4**, e08825 (2015).
